# Supplementary material for: Immune stealth-driven O2 serotype prevalence and potential for therapeutic antibodies against multidrug resistant Klebsiella pneumoniae
Source: Nat Commun. 2017 Dec 8;8:1991. doi: 10.1038/s41467-017-02223-7 (PMC5722860; doi:10.1038/s41467-017-02223-7)
Supplement: Supplementary file 1 — Supplementary Information [file 41467_2017_2223_MOESM1_ESM.pdf]

Supplemental Information

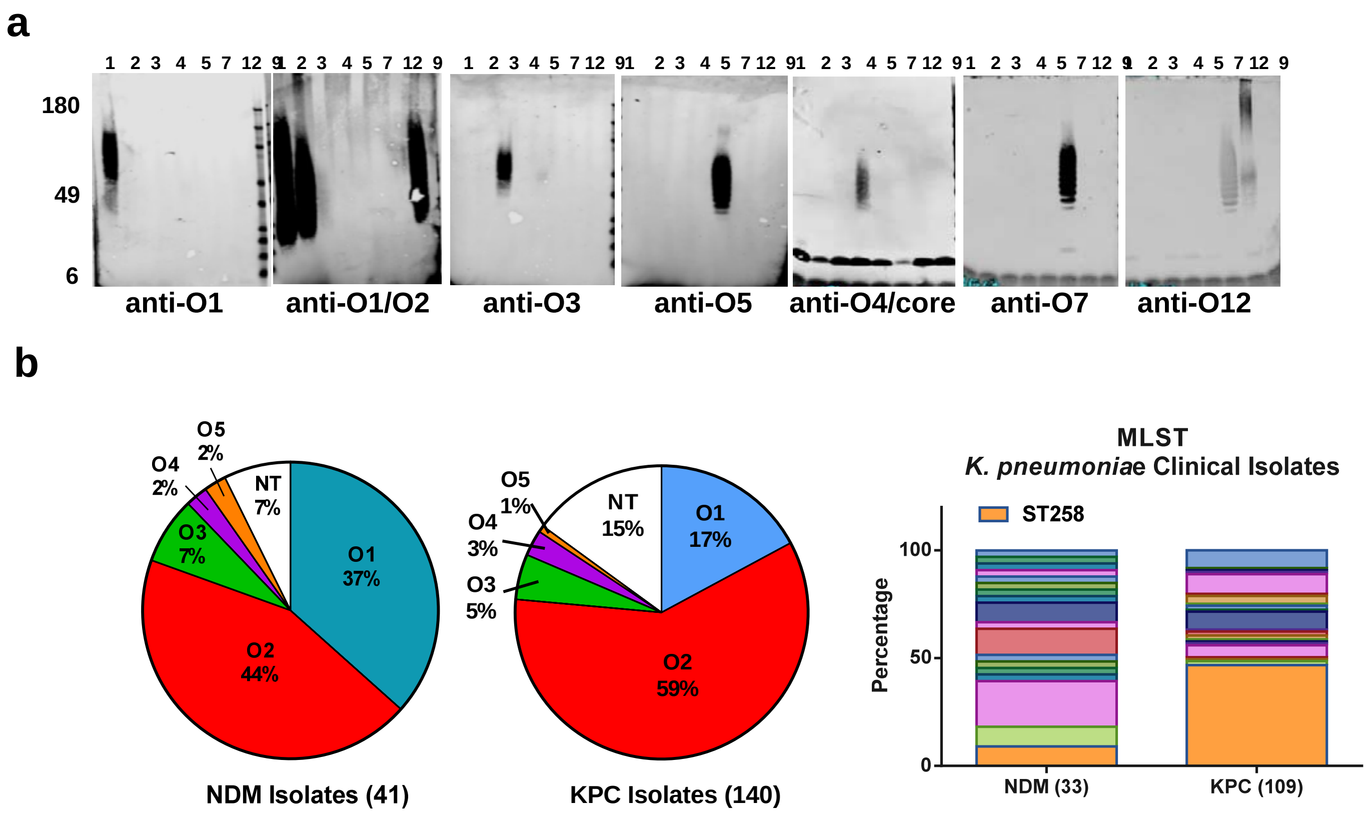

Supplementary Figure 1. Specificity of mAbs and breakdown of NDM and KPC isolates . a, Purified LPS (1 – 2 µg) from each of the LPS reference strains (listed in Supplementary Table 2) was run on a SDS-PAGE gel, transferred and probed using serotype-specific mAbs (O1, O2, O3, O4 and O5) or immunized mouse sera (O7 and O12). The specificity of the Ab is listed below the gel image, the lanes are labelled with the LPS serotype loaded in that lane. In our analysis, LPS serotype O9 was indistinguishable from O2. **b**, A subset of clinical isolates that tested positively by PCR for the carbapenem resistant gene family *ndm* (NDM isolates) or *kpc* (KPC isolates) were LPS serotyped. LPS O2 serotype was the most prevalent regardless of the resistance gene found in the isolate. In the MLST panel, a subset of the isolates from each pie chart was analyzed to determine ST type. ST258 is the predominant type in the KPC population but not in NDM isolates.

a

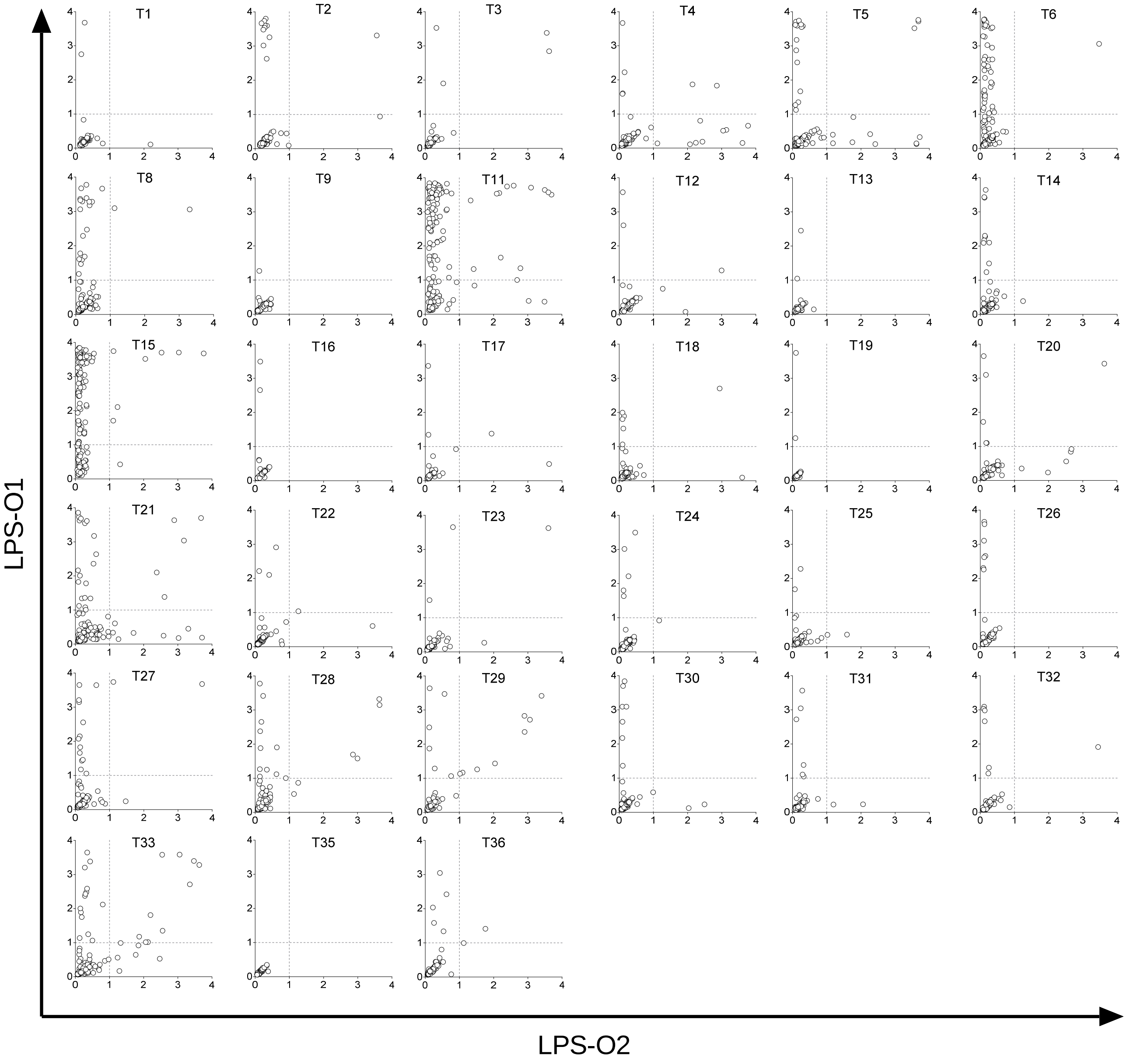
T1
T2
T3
T4
T5
T6
T8
T9
T11
T12
T13
T14
T15
T16
T17
T18
T19
T20
T21
T22
T23
T24
T25
T26
T27
T28
T29
T30
T31
T32
T33
T35
T36
LPS-O1
LPS-O2

b

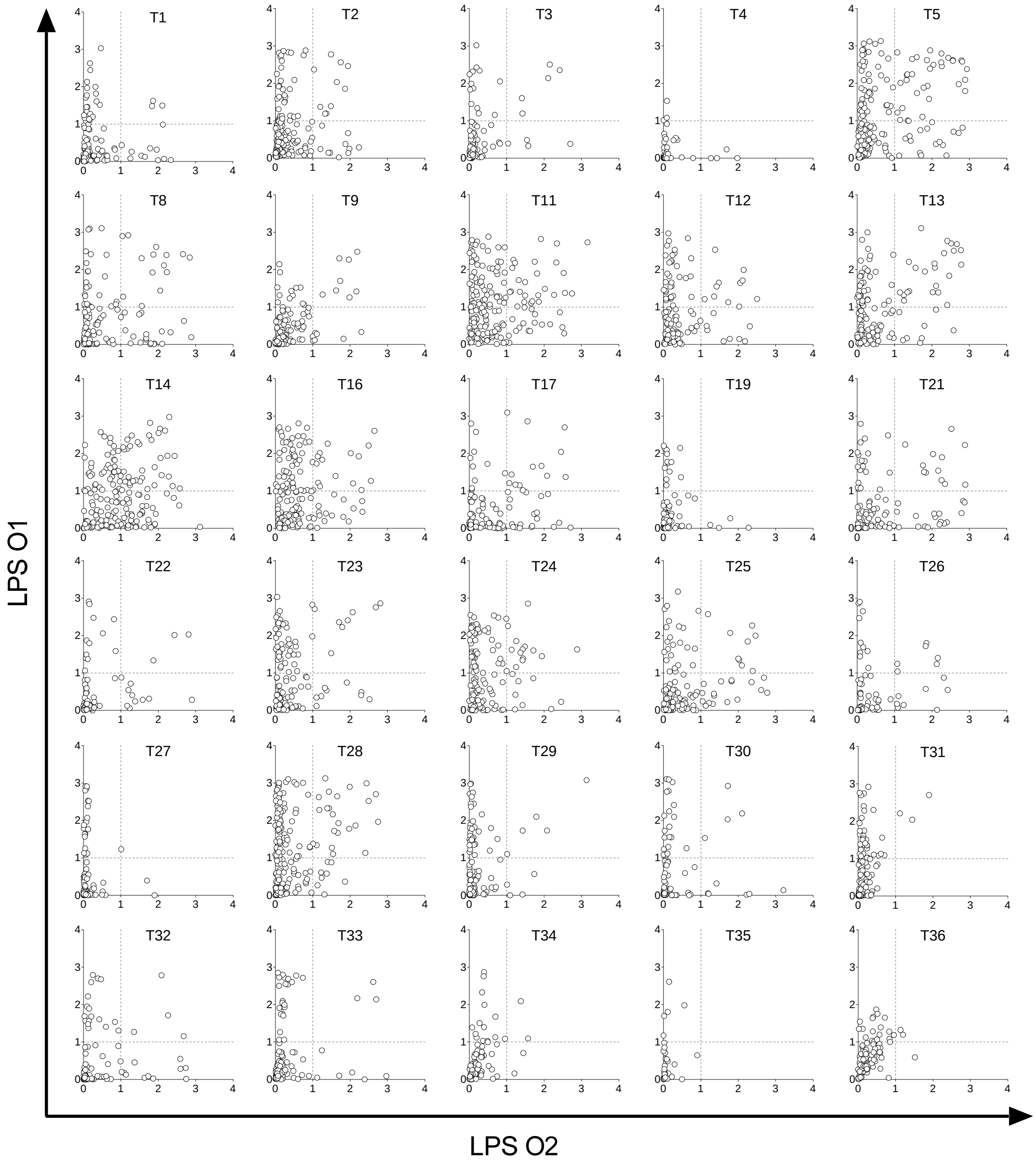

Supplementary Figure 2. **Clonal analysis of IgM and IgG memory B cells repertoires.** Analysis of antigen-specific repertoires (AMBRA) of tonsillar IgG (n=33) and IgM (n=30) memory B cells. Shown are the OD values at 405 nm of LPS O1 (upper left quadrant), LPS O2 (lower right quadrant) and O1/O2 (upper right quadrant) cross-reactive culture supernatants.

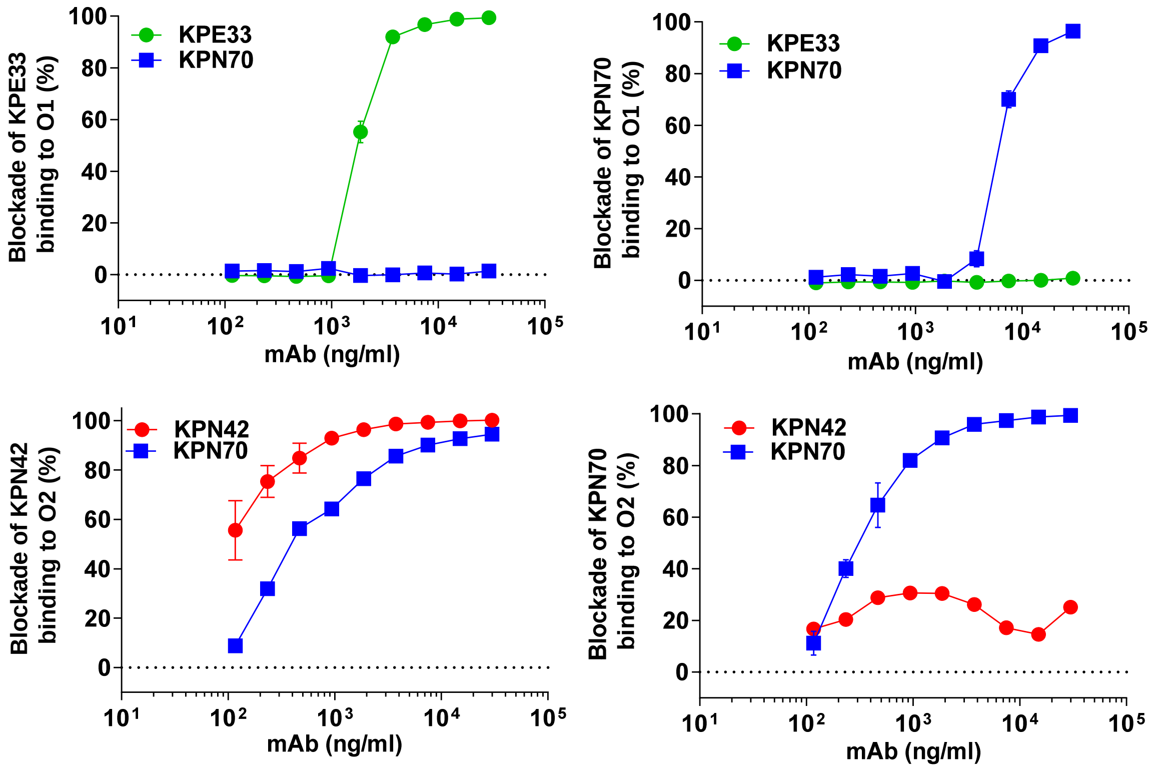

Supplementary Figure 3. Competitive binding of KPN70 with KPE33 and KPN42. Blocking of binding assays were performed as described in Materials and Methods to determine potentially shared epitopes between anti-O1 (KPE33) or anti-O2 (KPN42) mAbs with the cross-reactive mAb KPN70. Competition ELISAs were performed with O1 or O2 LPS coated plates using serially diluted non-labelled mAbs (green, blue or red symbols on graph) followed by the addition of biotinylated mAbs (indicated on Y axis). Error bars indicate s.d. at each data point.

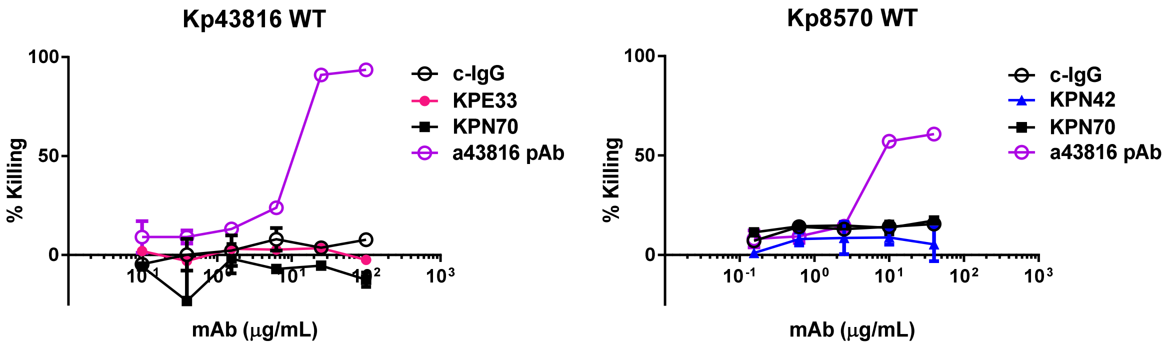

Supplementary Figure 4. OPK activity against encapsulated KP. OPK assays were performed as described in Material and Methods using WT encapsulated Kp43816 or 8570. Serially diluted mAbs were assessed for their ability to promote killing in the presence of phagocytic cell line HL-60 and baby rabbit serum as a complement source. Anti-43816 polyclonal IgG Ab (purified from heat-killed Kp43816 immunized rabbit sera, open circles) was used as a positive control. Error bars represent s.d. of each data point.

a

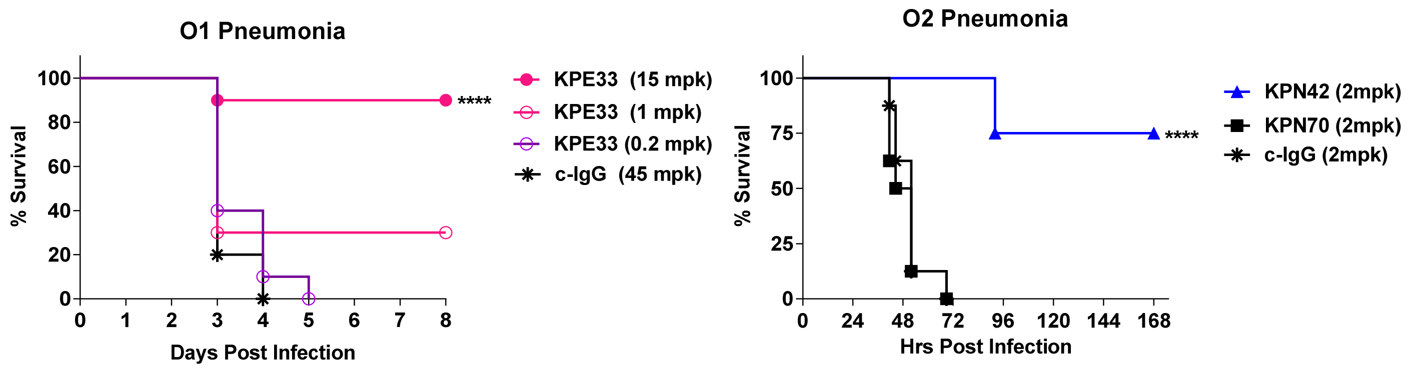

b

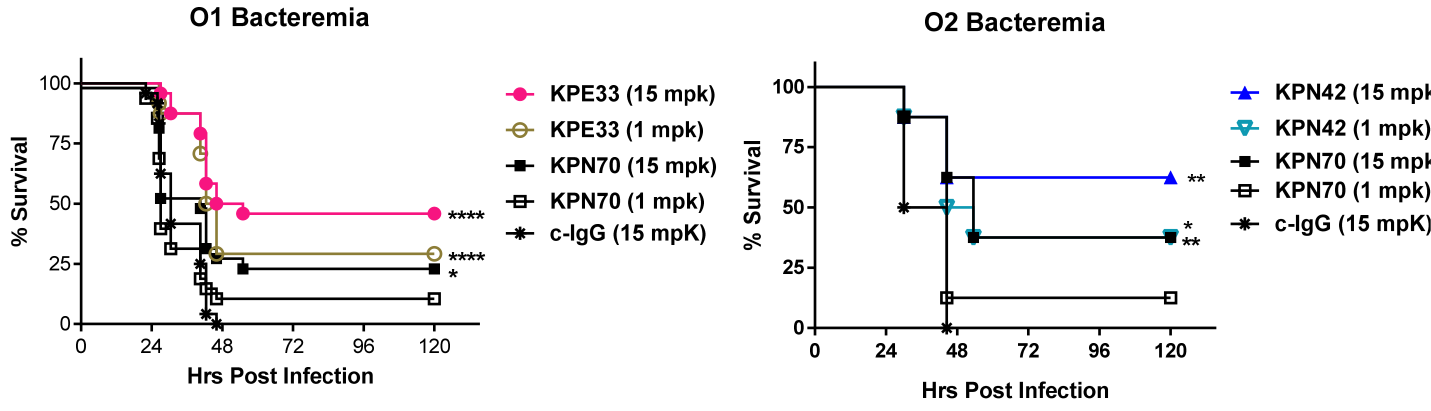

Supplementary Figure 5. Additional anti-LPS mAbs activity in vivo. a, C57BL/6 mice were infected intranasally as described in Materials and Methods with the O1 strain Kp8045 (5 x 10^4 CFU, K1 capsule serotype) or the O2 strain Kp977778 (3 x 10^8 CFU). mAbs were administered IV 1 h post infection at the indicated concentrations and survival was monitored for up to 8 days. **b**, C57BL/6 mice were infected IP with the O1 strain 1131115 (3 x 10^6 CFU) or the O2 strain 981842 (1 x 10^7 CFU). mAbs were administered IV 1 h post infection. Each graph represents at least 3 separate experiments. **** $p < 0.0001$, *** $p < 0.001$, ** $p < 0.01$, * $p < 0.05$ as determined by Mantel-Cox analysis of treated group versus control group (control mAb treated).

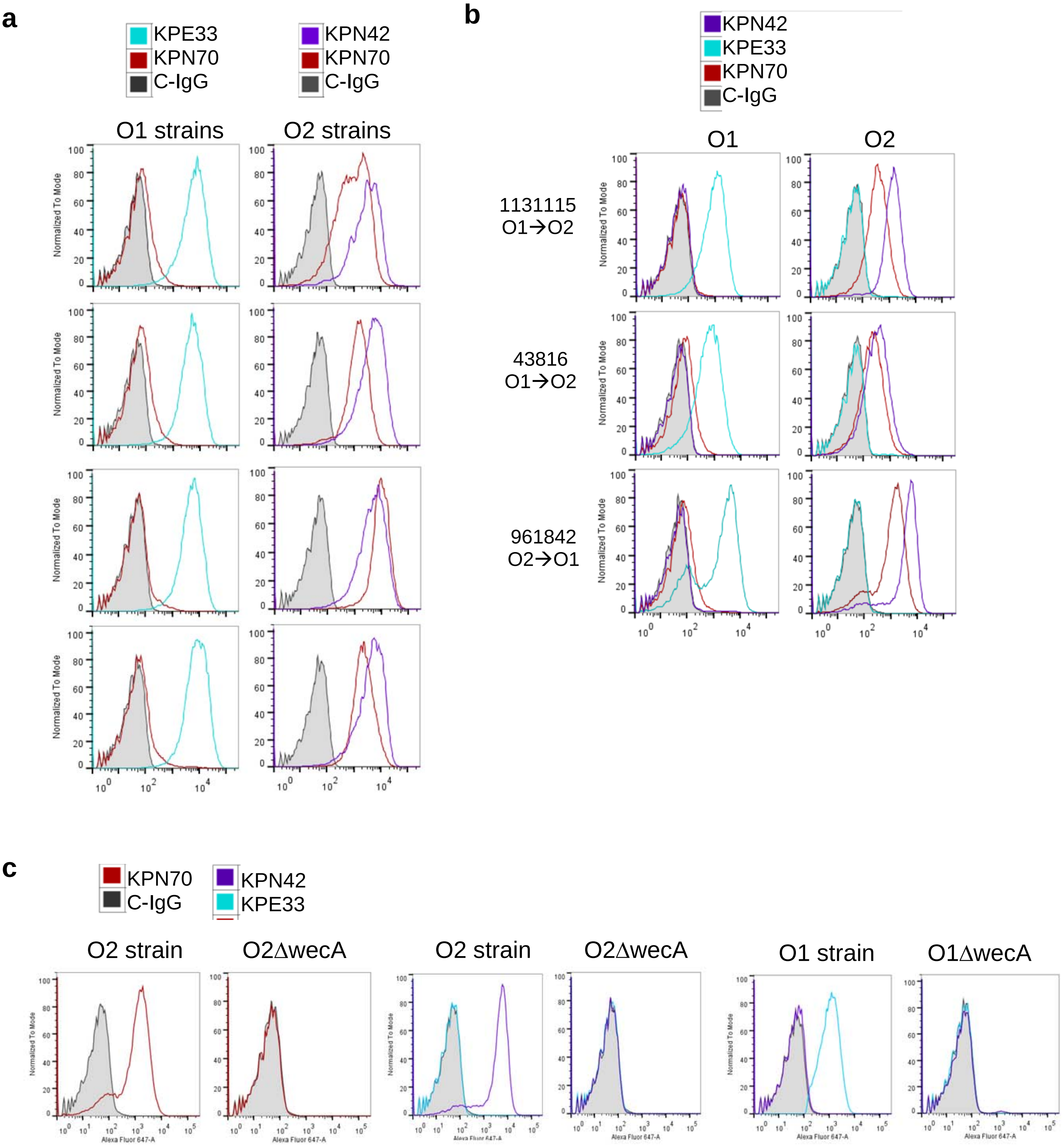
a
KPE33
KPN70
C-IgG
KPN42
KPN70
C-IgG
O1 strains
O2 strains
Normalized To Mode
b
KPN42
KPE33
KPN70
C-IgG
O1
O2
1131115
O1→O2
43816
O1→O2
961842
O2→O1
Normalized To Mode
c
KPN70
C-IgG
KPN42
KPE33
O2 strain
O2ΔwecA
O2 strain
O2ΔwecA
O1 strain
O1ΔwecA
Alexa Fluor 647-A

Supplementary Figure 6. **KPN70 binds preferentially to O2 strains**. **a**, A panel of O1 and O2 clinical isolates (4 each) were incubated in the presence of KPE33 or KPN42, KPN70 and a control IgG and analyzed by flow cytometry. **b**, O1 strains (Kp43816 and Kp1131115) were genetically converted to express O2 and both the O1 and O2 strains were analyzed by flow cytometry for binding to KPN70. The O2 strain 961842 was genetically converted to and O1 strain and analyzed for KPN70 binding. KPE33 and KPN42 were used as positive controls to ensure conversion of O serotype. Control IgG was used as a negative control. **c**, The O2 strain Kp961842 and an O-antigen deletion mutant (961842ΔwecA) were analyzed for KPN70, KPE33 and KPN42 binding. The O1 strain 1131115 and an O-antigen deletion mutant (1131115ΔwecA) were analyzed for KPE33 and KPN42 binding. Control IgG was used as a negative control.

Supplementary Table S1. Summary of *K. pneumoniae* isolates obtained for serotyping

	# of isolates	percent
Africa	35	5
Europe	270	38
Latin America	138	19
Middle East	39	6
North America	111	16
South Pacific/Asia	116	16
Total	709	100

	# of isolates	percent
Respiratory	409	57.7
Blood/peritoneal	65	9.2
GI	31	4.4
GU	99	14
Wound/skin	103	14.5
Total	707	99.7

Supplementary Table S2. Bacterial strains used in these studies

Strain	Source	LPS serotype	Resistance Category	Additional information
43816	ATCC	O1	S	K2 capsule serotype
43816Δcps	this study	O1	S	capsule deficient mutant; O1 LPS purified from this strain
43816ΔwbbYZ	this study	O2	S	D-gal II deletion mutant
5053	ATCC	O2	S	capsule deficient; O2 LPS purified from this strain
8570Δcps	Eurofin	O2	S	capsule deficient mutant; O2 LPS purified from this strain
1131115	IHMA	O1	CRE	
1131115ΔwbbYZ	this study	O2	CRE	D-gal II deletion mutant
961842	IHMA	O2	CRE	ST258
961842 p221	this study	O2	CRE	transfected with empty vector p221
961842 p308	this study	O1	CRE	transfected with plasmid p308 containing *wbbYZ* genes required for synthesis of D-gal II
961842ΔwecA	this study	none	CRE	O-antigen-deficient mutant
977778	IHMA	O2	CRE	ST258
8045	ATCC	O1	S	K1 capsule serotype
8561	Eurofin	O1	ESBL	
9178	NCTC	O3		capsule deficient; O3 LPS purified from this strain
9135	NCTC	O4		capsule deficient; O4 LPS purified from this strain
9181	NCTC	O5		capsule deficient; O5 LPS purified from this strain
9187	NCTC	O7		capsule deficient; O7 LPS purified from this strain
1205	NCTC	O9		capsule deficient; O9 LPS purified from this strain
11357	NCTC	O12		capsule deficient; O12 LPS purified from this strain

Supplementary Table S3. Plasma binding to O1 LPS and O2 in healthy and ICU donors

Plasma from 103 healthy donors and 6 ICU patients were tested in ELISA against to LPS-O1 or O2 (diluted 1:150). ELISA OD values are shown.

ID	O1	O2
691	3.746	0.931
692	1.358	1.13
693	1.84	0.9
694	1.157	0.987
695	1.272	0.752
696	0.539	0.462
697	1.029	0.5
698	0.564	0.353
699	2.353	1.696
700	1.071	0.435
701	0.525	0.273
702	1.433	0.538
703	1.254	1.028
704	1.158	0.3
705	3.496	0.29
706	1.329	0.636
707	1.153	0.313
708	1.053	0.729
709	2.055	0.775
710	0.597	0.187
711	1.199	0.761
712	0.556	0.833
713	0.352	0.375
714	0.845	0.382
715	0.676	0.406
716	0.957	0.789
717	1.941	0.456
718	0.567	0.176
719	1.239	0.323
720	1.611	0.407
721	0.543	0.187
722	2.021	0.27
723	0.549	0.248
724	0.815	0.371
725	1.3	0.372
726	1.606	0.384
727	1.858	0.576
728	0.239	0.281
729	2.18	2.74
730	0.625	0.265

ID	O1	O2
743	1.435	0.77
744	0.842	0.371
745	0.598	0.243
746	0.911	0.154
747	1.072	0.579
748	0.697	0.151
749	0.547	0.307
750	0.689	0.435
751	1.05	1.884
752	2.704	2.252
753	1.22	0.218
754	1.597	0.129
755	0.673	0.355
756	2.289	2.137
757	1.346	0.814
758	0.962	0.235
759	1.407	0.386
760	1.389	0.546
761	0.708	0.218
762	0.959	1.176
763	0.912	0.551
764	0.555	0.656
765	1.328	0.476
766	1.294	0.288
767	0.889	0.236
768	0.293	0.142
769	1.646	0.891
770	0.298	0.223
771	0.519	0.146
772	0.354	0.146
773	0.62	0.183
774	0.541	0.343
775	1.157	0.415
776	0.292	0.14
777	0.651	0.501
778	1.337	0.802
779	0.615	0.277
780	3.417	2.157
781	0.938	0.424
782	1.374	0.382

ICU plasma	O1	O2
KL2T2	1.752	1.254
KL6T2	3.657	3.771
KL6T3	3.685	3.69
KL7T2	2.936	2.785
KL5T2	3.962	2.632
KL10T2	1.457	1.205
KL9T2	0.623	0.369

**731**	0.306	0.236
**732**	0.791	0.299
**733**	0.599	0.417
**734**	0.872	0.368
**735**	0.795	0.31
**736**	0.423	0.314
**737**	0.753	0.353
**738**	1.027	0.411
**739**	1.519	0.796
**740**	0.963	0.116
**741**	0.443	0.105
**742**	0.795	0.618

**783**	1.187	0.814
**784**	0.538	0.157
**785**	1.098	0.933
**786**	0.804	0.525
**787**	0.715	0.23
**788**	1.156	0.337
**789**	3.151	0.451
**790**	1.399	0.217
**791**	0.895	0.546
**792**	1.943	0.286
**793**	2.43	1.47

0 - 0.8
0.801 - 1.5
1.51 - 4
